# Establishment of *in vitro* regeneration system and molecular analysis of early development of somatic callus in *Capsicum chinense* and *Capsicum baccatum*

**DOI:** 10.3389/fpls.2022.1025497

**Published:** 2022-11-17

**Authors:** Huangying Shu, Yu Zhang, Chengyao He, Muhammad Ahsan Altaf, Yuanyuan Hao, Daolong Liao, Lin Li, Caichao Li, Huizhen Fu, Shanhan Cheng, Guopeng Zhu, Zhiwei Wang

**Affiliations:** ^1^ Key Laboratory for Quality Regulation of Tropical Horticultural Crops of Hainan Province, School of Horticulture, Hainan University, Haikou, China; ^2^ Sanya Nanfan Research Institute, Hainan University, Sanya, China; ^3^ Hainan Yazhou Bay Seed Laboratory, Sanya, China; ^4^ Institute of Vegetables, Hainan Province Academy of Agricultural Sciences, Haikou, China

**Keywords:** *Capsicum chinense*, *Capsicum baccatum*, somatic callus, *in vitro* regeneration transcriptome, histology, WGCNA

## Abstract

Regeneration is extremely important to pepper genetic development; however, the molecular mechanisms of how the callus reactivates cell proliferation and promotes cell reprogramming remain elusive in pepper. In the present study, *C. baccatum* (HNUCB81 and HNUCB226) and *C. chinense* (HNUCC22 and HNUCC16) were analyzed to reveal callus initiation by *in vitro* regeneration, histology, and transcriptome. We successfully established an efficient *in vitro* regeneration system of two cultivars to monitor the callus induction of differential genotypes, and the regenerated plants were obtained. Compared to *C. chinense*, there was a higher callus induction rate in *C. baccatum*. The phenotype of *C. baccatum* changed significantly and formed vascular tissue faster than *C. chinense*. The KEGG enrichment analysis found that plant hormone transduction and starch and sucrose metabolism pathways were significantly enriched. In addition, we identified that the *WOX7* gene was significantly up-regulated in HNUCB81 and HNUCB226 than that in HNUCC22 and HNUCC16, which may be a potential function in callus formation. These results provided a promising strategy to improve the regeneration and transformation of pepper plants.

## Introduction

A genetic transformation is an important tool for functional genome research and an effective technique for crop breeding (Altpeter et al., 2016). The method of plant transformation depends on plant tissue culture *in vitro*, which is an initial step of plant transformation. However, *Capsicum* spp. is a recalcitrant plant that has some obstacles in tissue culture and organ differentiation *in vitro* (Kothari et al., 2010), which is a major bottleneck in pepper transformation. Therefore, establishing an efficient regeneration system is extremely vital for pepper genetic development.

Plants have a complex physiological and molecular process for dealing with regeneration (Ikeuchi et al., 2013). Many regulated genes have been extensively characterized, which play an essential role in the regeneration of plants. For example, previous studies revealed that phytohormones play a key role in callus induction, and auxin-related genes are regarded as an important regulator in regeneration, including *Gretchen Hagen 3* (*GH3*), *small auxin upregulated RNA* (*SAUR*), and *Auxin/Indole-3-Acetic Acid* (*AUX/IAA*) (Méndez-Hernández et al., 2019). The transcription factor (TFs) *WUSCHEL* (*WUS*) was strongly expressed in some callus. It was found that overexpression of *WUS* generated callus and somatic embryos in *Arabidopsis.* Moreover, *WUS* interactions with *CLV3* establish a feedback loop between the stem cells (Zuo et al., 2002; Iwase et al., 2011). Interestingly, multiple TFs have been postulated involving somatic callus competence, including *LATERAL ORGAN BOUNDARIES DOMAIN* (*LBD*) (Fan et al., 2012), *WOUND INDUCED DEDIFFERENTIATION1* (*WIND1*), *bud regeneration enhancer 1* (*ESR1*) (Iwase et al., 2017), *Baby Boom* (*BBM*) (Heidmann et al., 2011; Chen et al., 2022), and *WUSCHEL RELATED HOMEOBOX (WOX)* (Ikeuchi et al., 2021). In addition, studies have shown that DNA methylation and histone might be both associated with gene expression, and this mechanism could control cell differentiation and dedifferentiation (Ikeuchi et al., 2015).

Pepper (*Capsicum* spp.) is one of the most important crops and is widely applied for seasoning, pharmaceuticals, and cosmetics (Kothari et al., 2010). Pepper is cultivated worldwide because of its superior adaptability (Qin et al., 2014). The research on the pepper reference genome has accelerated the identification of genes related to important biological processes (Kim et al., 2017). A large number of studies have been widely performed to optimize the protocol of callus regeneration and transformation of various pepper species (Lee et al., 2004; Sanatombi and Sharma, 2008; Sanatombi and Sharma, 2012). However, as pepper is highly recalcitrant for regeneration and transformation, the underlying mechanism remains unclear. Therefore, it is necessary to reveal the key regulatory network of pepper regeneration.

This study constructed the regeneration system of *C. baccatum* and *C. chinense* and obtained the regenerated plants by *in vitro* regeneration. To find key regulated genes to the phase change of cotyledon petiole explants and callus initiation in pepper, we sought to perform histologically, and transcriptome analysis of four inbred lines with different organogenesis rates. In the process of *in vitro* regeneration, we found a phase transition phenomenon of pepper explants. Besides, we identified some enrichment pathways involved in organogenesis. Several candidate genes were described related to auxin transport, metabolism, and cell development, which have been previously identified to be associated with regeneration. Our findings provide an important molecular framework that supplies in-depth insight into the genetics of pepper regeneration.

## Results

### Establishment of *in vitro* regeneration system by exogenous hormones

**Figure 1:**
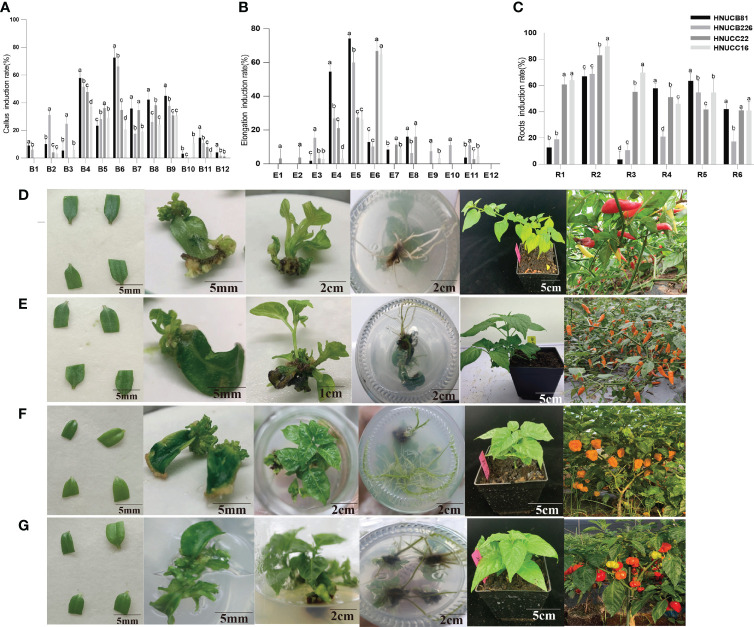
Construction of *in vitro* regeneration system. **(A)** callus induction rate, **(B)** elongation induction rate, and **(C)** roots induction rate of the four inbred lines. Abscissa indicated the media name. The regeneration induction growth status of pepper of **(D)** *C. baccatum* (HNUCB81), **(E)** *C. baccatum* (HNUCB226), **(F)** *C. chinense* (HNUCC16), and **(G)** *C. chinense* (HNUCC22), respectively.

6-Benzylaminopurine (6-BA) and 3-Indoleacetic acid (IAA) were applied to detect the optimum concentration of exogenous hormones for somatic callus induction. Cotyledons with petioles of 15-20 days seedlings were used as explants. In media B6 (MS+ 13.32µM 6-BA+ 2.85µM IAA), the induction rates of HNUCB81 and HNUCB226 were the highest (72.58% and 66.22%), and the best induction media for HNUCC22 and HNUCC16 was B4 (MS+ 13.32µM 6-BA+ 0.57µM IAA), and the induction rates were 48% and 37%, respectively. Compared with *C. chinense* in the B1 media, *C. baccatum* induction rate was slightly higher (57.89% and 52.86%) (**Figure 1A** and **Supplementary Table S1**). Furthermore, we induced adventitious buds by supplementing cytokinin TDZ alone, but the induction effect was poor.

We further found that the application of 6-BA + IAA with differential GA_3_ led to an increased elongation rate. The best elongation induction rates of HNUCC22 and HNUCC16 were 66.78% and 64.61% in E6 media (MS + 13.32µM 6-BA + 5.71µM IAA + 11.55µM GA_3_), respectively. While HNUCB81 and HNUCB 226 could obtain higher elongation induction rates (74.27% and 60%) in media E5 (13.32µM 6-BA + 5.71µM IAA + 5.78µM GA_3_). Besides, a high concentration of 6-BA resulted in a low elongation induction rate (**Figure 1B** and **Supplementary Table S2**).

The rooting induction rate of supplemented IAA was the optimum among the four inbred lines. In media R2 (1/2MS + 1.14µM IAA), the highest rooting induction rate of the four inbred lines was obtained, which was over 60%. In addition, adding a certain amount of NAA can also induce a good rooting induction rate in *C. chinense*. On the contrary, it could obtain a quite low induction rate in *C. baccatum* (**Figure 1C** and **Supplementary Table S4**). Therefore, we constructed the regeneration systems of two cultivars (**Figures 1D–G**). Further studies were carried out through the comparison of different genotypes.

### Phenotypic and histology evaluation of pepper

**Figure 2:**
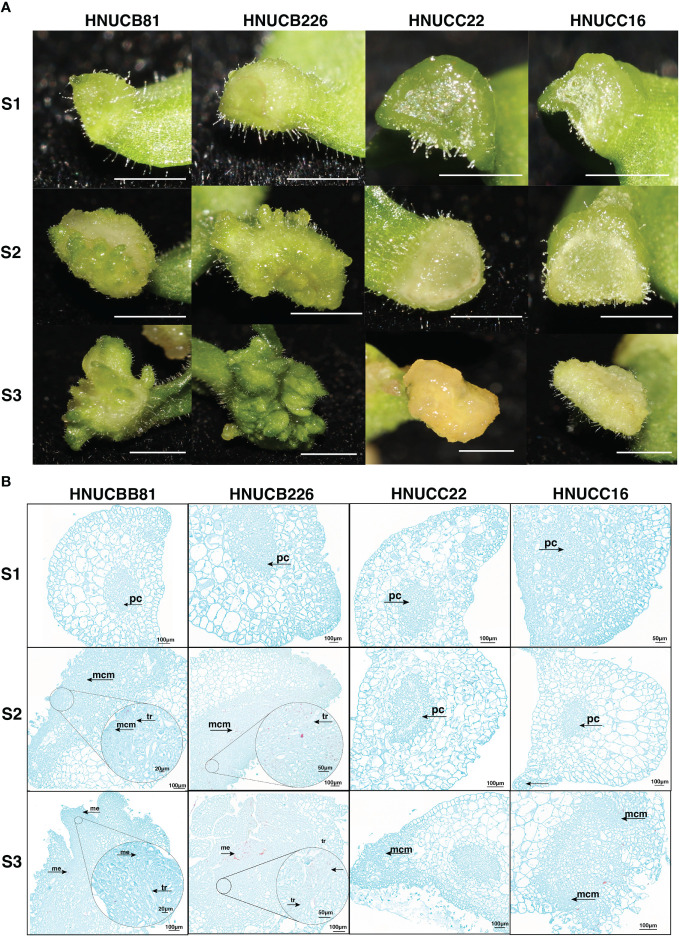
Phenotypic of pepper callus at three development stages. **(A)** The growth status of the embryonic callus of four genotypes, Bar=2mm; **(B)** The anatomical morphology of callus at different stages. (**tr**: vessels, **pc**. Parenchyma cells, **mcm**: Meristematic cell mass, and **me**: Meristemoid.).

Compared with elongation and root induction rate, *C. chinense* callus differentiating rate was lower than *C. baccatum*. We investigated the phenotypic differences between *in vitro* plantlets at an early stage. For *C. baccatum* genotypes HNUCB81 and HNUCB226, a few adventitious-like buds could be observed at the cotyledon petiole tip at the S2 stage. Some plantlet-like structures were growing at the S3 stage. HNUCC22 and HNUCC16 produced only some callus and did not format adventitious during the three stages (**Figure 2A**).

To gain insight into the morphological variation of four lines, the histological analysis examined the organogenesis of pepper regeneration. It was found that the differentiation of parenchyma cells near the epidermis was dedifferentiated and restored the ability to divide under the stimulation of phytohormones from *C. baccatum*. Compared with *C. chinense*, the cells of HNUCB81 and HNUCB226 were arranged more densely during the S2 and S3 stages. However, loosely packed with large cellular spaces of HNUCC22 and HNUCC16. Furthermore, we found that the bud primordia produced by pepper tissue culture originated from several layers of parenchyma cells with the callus (**Figure 2B**).

### Transcriptome sequencing of pepper callus

We sequenced the transcriptome of the embryonic callus at three different stages to find out how these regulated genes affect pepper shoot regeneration in the early stages of growth. A total of 36 libraries were established by the transcriptome sequencing of different callus levels of four genotypes and by evaluating the repeatability of the data (**Supplementary Figure S1**). About 151.89 million clean reads were generated. The average GC content was 42.21%, and the Q30 was ~92.56%. Interestingly, the proportion of 92.68% to 84.03% sequencing reads mapped to the *C. chinense* reference genome, and the remaining reads ranging from 82.25% to 94.07% were uniquely mapped to the four genotypes (**Supplementary Table S4**).

### Differentially expressed genes of different genotypes

**Figure 3:**
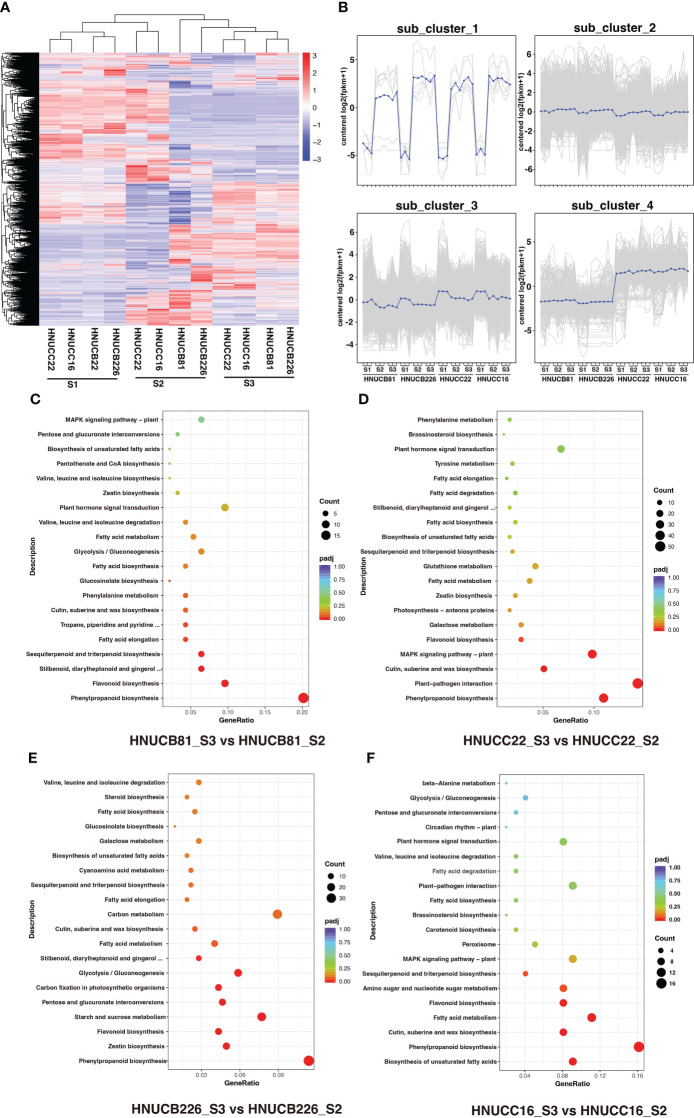
DEGs between four inbred lines at differential stages. **(A)** Heatmap and cluster analysis expression level of DEGs. **(B)** Magnified regions of 4 subclusters in all DEGs, blue line indicate consensus of each subcluster. **(C-F)** The top 20 KEGG pathway terms for the DEGs.

*C. baccatum* and *C. chinense* demonstrated the greatest differences in S2 and S3; however, they suggested a similar expression profile in the S1 (**Figure 3A**). Four cluster profiles were indicated, including 7, 17442, 9322, and 1027, respectively (**Figure 3B**). Moreover, the Venn diagram showed that the common DEGs were higher than the specific DEGs among four genotypes (**Supplementary Figure S2**). Kyoto Encyclopedia of Genes and Genomes (KEGG) analyses. **Figures 3C-F** shows several pathways that play a role in callus regeneration, such as phenylpropanoid biosynthesis, flavonoid biosynthesis, starch and sucrose metabolism.

### Analysis of co-expressed gene networks

**Figure 4:**
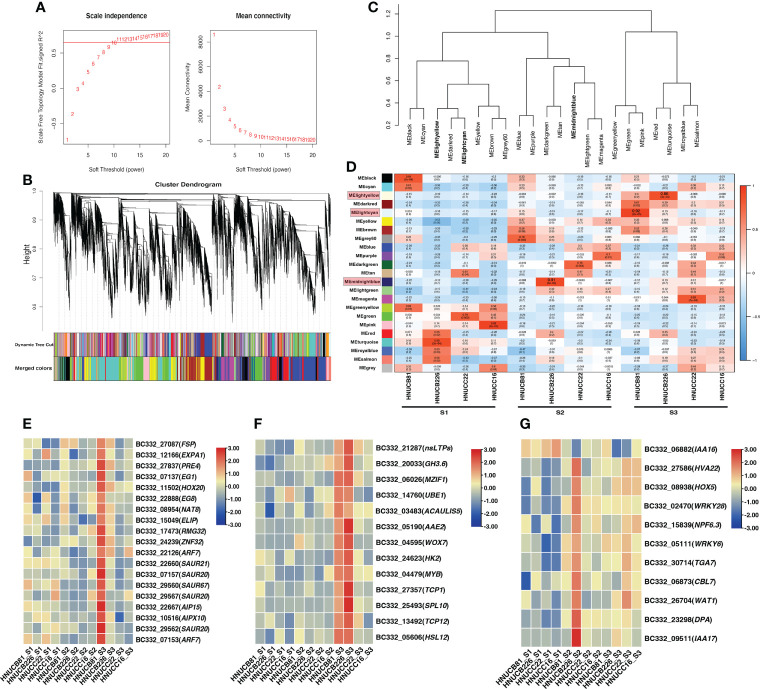
Weight-gene co-expression network analysis of pepper callus regeneration. **(A)** Soft-threshold of pepper callus regeneration. **(B)** Hierarchical clustering presents 23 modules having co-expressed genes. **(C)** Hierarchical cluster dendrogram of eigengenes. **(D)** Heatmap presents the relationship between the module and developmental stages. Red indicated a positive correlation and blue indicates a negative correlation. The heat map indicates gene expression of DEGs in the lightcyan module **(E)**, lightyellow module **(F)**, and midnightblue module **(G)**. The scale shows FPKM values.

Distributed to twenty-three modules to reveal the correlation between the genes at different stages based on the matrix set as soft-thresholding power =10 (**Figure 4A**). The modules were presented in a hierarchical clustering dendrogram, in which different colors indicate different modules; each branch represents an individual gene (**Figure 4B**). After filtering. lightcyan, lightyellow, and midnightblue modules were identified, which showed high-association specificity in S2 or S3 (**Figures 4C, D**).

**Figure 5:**
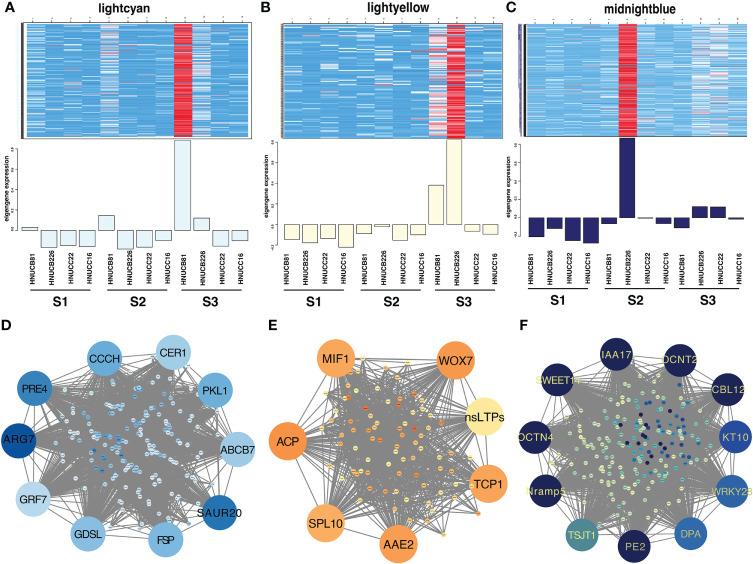
Gene network and candidate genes. Heat map showing the relative FPKM of each gene from lightcyan module (*r* ^2^ = 0.92, *p* =2e-05) **(A)**, lightyellow module (*r* ^2^ = 0.89, *p* =3e-04) **(B)**, and midnightblue module (*r* ^2^ = 0.91, *p* =4e-05) **(C)**. The correlation network of the hub gene of lightcyan module **(D)**, lightyellow module **(E)**, and midnightblue module **(F)**.

Most of the selected somatic regeneration-related genes were significantly up-regulated with a higher organogenesis rate at S2 and S3 (**Figures 4E-G**). *HOX20* was specifically expressed in HNUCB81 but not expressed in the S1 stage. *WOX7* was mainly expressed in HNUCB81 and HNUCB226 at S3 but not in S1. *AAE2* and *SPL10* were specifically expressed in *C. baccatum* at the S3, and the highest expression was found in HNUCB226 (**Figure 4F**). *IAA16* was specifically at the S1 but had lower expression at the S3 (**Figure 4G** **)**. A total of 200, 107, and 230 genes were included in three modules, respectively (**Supplementary Table S5**). The 26 genes that are called “hub genes” were shown in **Figure 5** and **Table 1** as part of the study.

**Table 1:** Information of hub genes.

Module	Gene name	kWithin	Description
lightcyan	ABCB7	75.44	ABC transporter B family member 7
lightcyan	CCCH	72.44	Zinc finger CCCH domain-containing protein 49
lightcyan	PKL1	72.07	Leucine-rich repeat receptor-like protein kinase PXL1
lightcyan	GRF7	70.56	Growth-regulating factor 7
lightcyan	GDSL	69.34	GDSL esterase/lipase
lightcyan	PRE4	68.68	Transcription factor PRE4
lightcyan	FSP	67.19	Fruit-specific protein
lightcyan	CER1	65.68	Protein CER1-like 1
lightcyan	SAUR20	54.63	Auxin-responsive protein 20
lightcyan	ARG7	32.99	Indole-3-acetic acid-induced protein 7
lightyellow	AAE2	37.99	putative acyl-activating enzyme 2
lightyellow	SPL10	34.24	Squamosa promoter-binding-like protein 10
lightyellow	nsLTPs	33.65	Non-specific lipid-transfer protein
lightyellow	TCP1	32.55	Transcription factor TCP1
lightyellow	WOX7	31.81	WUSCHEL-related homeobox 7
lightyellow	MIF1	31.058	Mini zinc finger protein 1
lightyellow	ACP	30.42	Acyl-[acyl-carrier-protein] desaturase
midnightblue	OCTN2	78.03	Organic cation/carnitine transporter 2
midnightblue	OCTN4	75.79	Organic cation/carnitine transporter 4
midnightblue	IAA17	74.33	Auxin-responsive protein 17
midnightblue	DPA	72.67	Transcription factor-like protein DPA
midnightblue	Nramp5	72.15	Metal transporter Nramp5
midnightblue	WRKY28	71.63	putative WRKY transcription factor 28
midnightblue	KT10	68.38	Potassium transporter 10
midnightblue	SWEET14	67.62	Bidirectional sugar transporter SWEET14
midnightblue	TSJT1	65.39	Stem-specific protein TSJT1

### Enrichment analysis of co-expression

**Figure 6:**
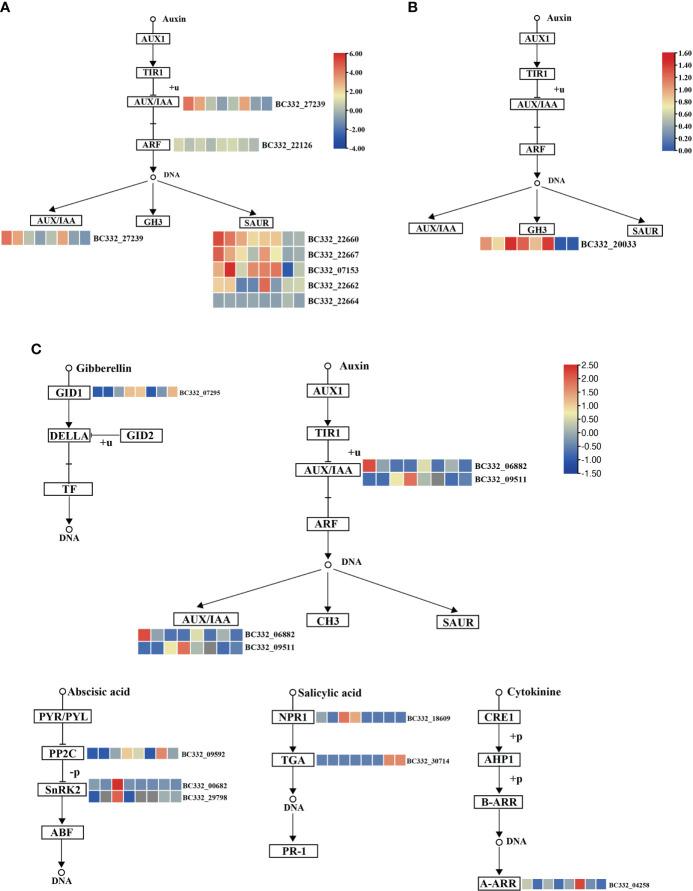
Pepper regeneration candidate DEGs in plant hormone signal transduction pathway. **(A)** lightcyan module, **(B)** lightyellow module, and **(C)** midnightblue module. The columns in the heatmap were displayed as HNUCB81_S3 *vs* HNUCC22_S3, HNUCB81_ S3 *vs* HNUCC16_ S3, HNUCB226_S3 *vs* HNUCC22_S3, HNUCB226_S3 *vs* HNUCC16_S3, HNUCB81_S3 *vs* HNUCB81_S2, HNUCB226_S3 *vs* HNUCB226_S2, HNUCC22_S3 *vs* HNUCC22_S2, and HNUCC16_S3 *vs* HNUCC16_ S2 of pepper regeneration from left to right, respectively.

**Figure 7:**
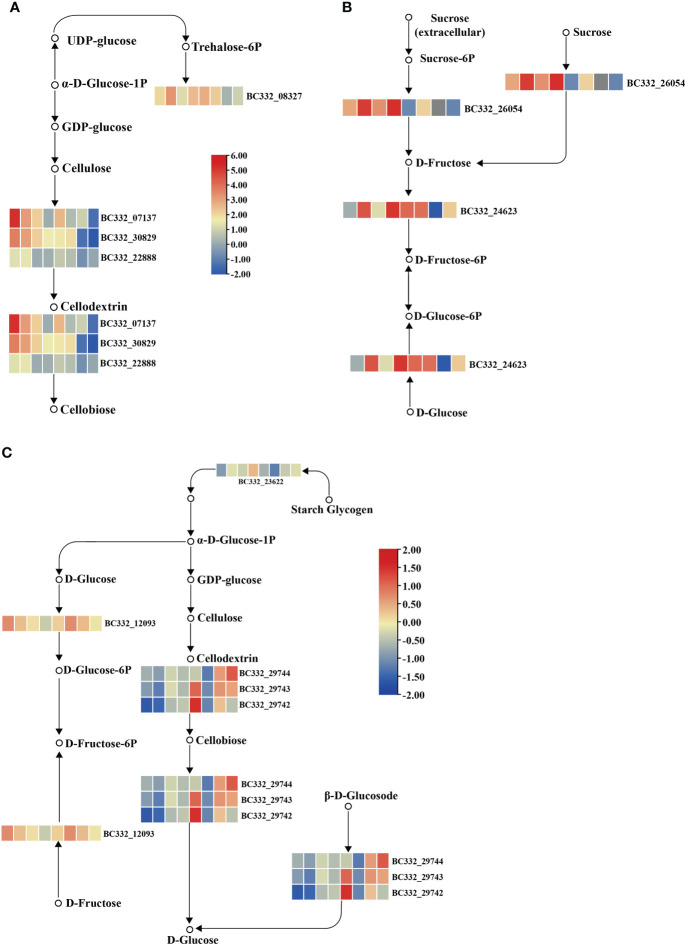
Pepper regeneration candidate DEGs in starch and sucrose metabolism pathway. **(A)** lightcyan module, **(B)** lightyellow module, and **(C)** midnightblue module. The columns in the heatmap were displayed as HNUCB81_S3 *vs* HNUCC22_S3, HNUCB81_S3 vs HNUCC16_S3, HNUCB226_ S3 *vs* HNUCC22_S3, HNUCB226_S3 *vs* HNUCC16_S3, HNUCB81_S3 *vs* HNUCB81_S2, HNUCB226_S3 *vs* HNUCB226_S2, HNUCC22_S3 *vs* HNUCC22_S2, and HNUCC16_S3 *vs* HNUCC16_ S2 of pepper regeneration from left to right, respectively.

We conducted KEGG on three modules. Interestingly, plant hormone signal transduction and starch and sucrose metabolism were significantly enriched (**Supplementary Table S6**). In the plant hormone signal transduction pathways, five DEGs were up-regulated in HNUCB81_S3 but down-regulated in HNUCC22_S3 and HNUCC16_S3 of the lightcyan module, which all belong to the auxin-inducible TF family (**Figure 6**). A total of four and two DEGs were involved in the starch and sucrose metabolism pathway between the lightcyan and lightyellow module, and all of the genes were up-regulated in *C. baccatum* at the S3. However, the results of the midnightblue module are inconsistent with the other two modules, suggesting that these pathways may not have been activated at the S2 (**Figure 7**). Furthermore, Gene Ontology (GO) describes the molecular and biological functions of the genes involved in regeneration. DEGs were distributed in the molecular function (MF), cellular component (CC), and biological process (BP) categories, respectively (**Supplementary Figure S3** and **Supplementary Table S7**).

### TFs involved in callus induction

Previous studies have indicated that TFs play a key role in callus formation by regulating cell differentiation and proliferation (Ikeuchi et al., 2019). We further analyzed the TFs between four inbred lines; 737, 156, 116, and 610 TFs were identified in HNUCC22, HNUCC16, HNUCB81, and HNUCB226 between S2 and S3, respectively. Pkinase, p450, NB-ARC, WRKY, LRRNT_2, HLH, and MYB TFs account for a large proportion (**Supplementary Table S8**).

### Validation of DEGs expression by qRT-PCR

**Figure 8:**
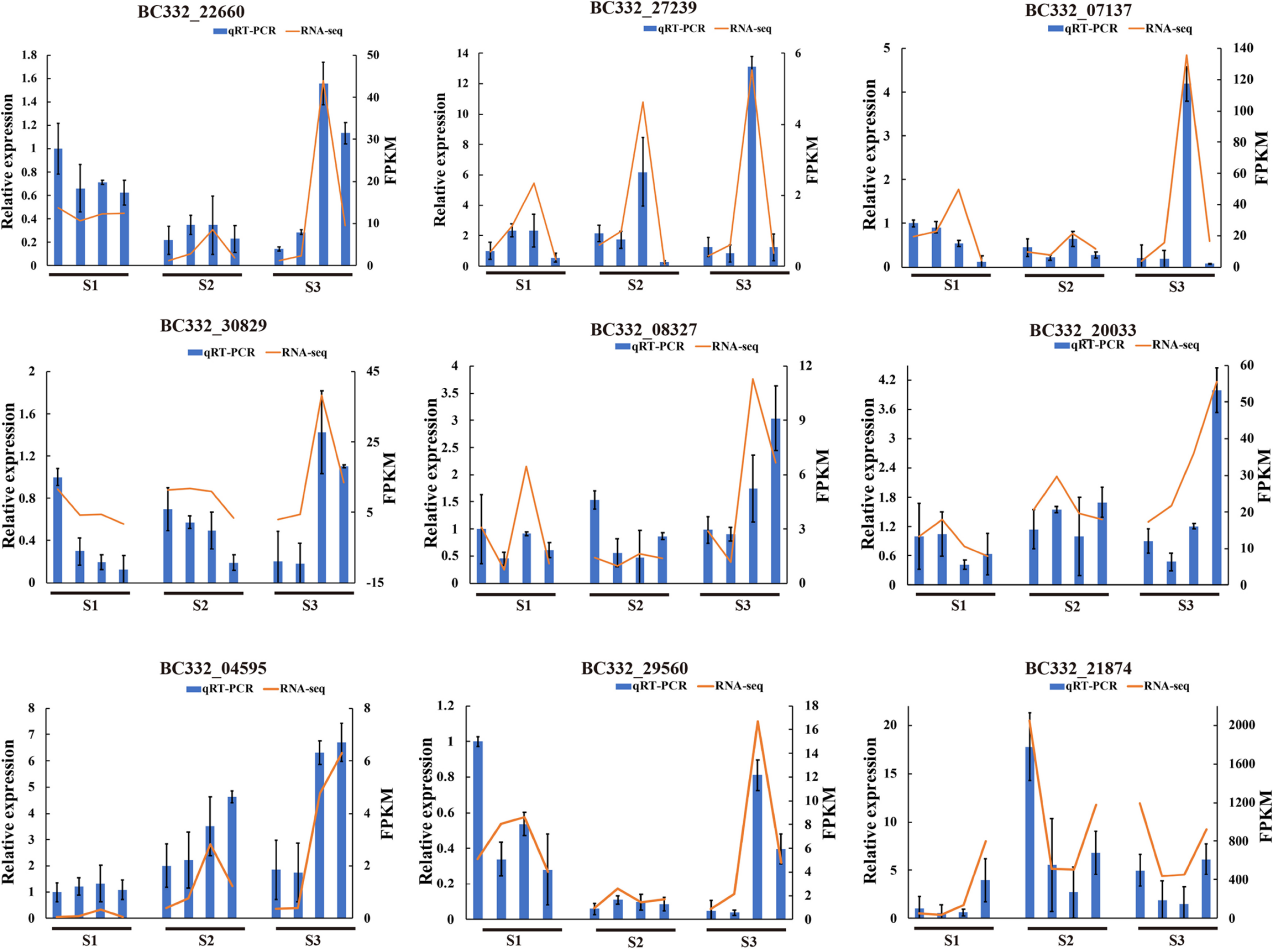
Correlation between qRT-PCR and RNA-seq based on differentially expressed genes of enrichment pathways at three differentiation stages. HNUCC22, HNUCC16, HNUCB81, and HNUCC226 are represented from left to right at three stages, respectively.

To validate the transcriptome sequencing data, 9 DEGs involved in enrichment pathways were selected for qRT-PCR. The results suggested that the expression patterns of qRT-PCR were the same as those of RNA-seq data, revealing that the RNA-seq data is reliable (**Figure 8**).

## Discussion

Pepper is a highly genotype-dependent, and recalcitrant plant, which leads to the development of *in vitro* regeneration system being relatively slow. Traditional breeding methods are being gradually updated. Plant biotechnology, including tissue culture and genetic engineering, is gradually becoming an important breeding method to promote the improvement of pepper breeding. It is well known that several genes are involved in explant regeneration, including *WOX*, *BBM*, *LEC*, and *WUS* (Zhao et al., 2022). Our results indicated that phytohormones play an important role in callus formation by regulating the expression of regeneration-related genes. In addition, WGCNA analysis identified many genes linked to plant hormone regulation, which has a positive correlation with cell differentiation. This study illustrated how pepper conveys a diverse range of signals to regulate the early regeneration process in pepper.

### Study on regeneration system of pepper *in vitro*

Auxin and cytokinin regulate cell division, callus differentiation, and somatic embryogenesis (Pinto et al., 2011). It was reported that the percentage of bud-forming explants was the highest under the induction of 3.4 µM TDZ (Santanabuzzy et al., 2005). Venkataiah reported that the highest induction rate was obtained for *C. baccatum* when supplemented with 2,4-D and Kinetin (Venkataiah et al., 2016). This study constructed the *in vitro* regeneration systems of four inbred lines of pepper. *C. chinense* and *C. baccatum* somatic callus were produced in the presence of 6-BA and IAA. However, there are distinct differences between the two cultivated species. Our results showed that supplementation with GA_3_ markedly promoted elongation, but the higher levels of 6-BA inhibited the induction of elongation. Furthermore, we observed that the application of IAA significantly increased the induction of roots in *C. chinense*. In the induction media of 1.07µM IAA, the ability of pepper to induce rooting was the most significant. These results laid the foundation for genetic transformation and molecular-assisted breeding of pepper.

### Plant hormone’s effects on pepper regeneration

Previous studies have reported that plant hormones regulate many aspects of plant growth and development (Su et al., 2011). Auxin, ethylene, and abscisic acid (ABA) hormones are considered to play a positive role in organogenesis and regeneration (Méndez-Hernández et al., 2019). The auxin biosynthesis-related gene *AtSAUR36* has been linked to plant cell expansion (Hou et al., 2013). A recent study revealed that *ZmSAUR15* has an adverse effect on the embryogenic callus (ECs) formation of maize (Wang Y. et al., 2021). Our study demonstrated that the plant hormone transduction and biosynthesis genes were involved in the process of pepper regeneration potential. We found that most of the auxin-related genes were up-regulated in *C. baccatum* but down-regulated in *C. chinense*, indicating that auxin transduction was the key factor of callus regeneration. Moreover, we performed qRT-PCR, showing that the expression of *GH3.6* (*BC332_20033*) and *SAUR67* (*BC332_29560*) increased dramatically at S3, suggesting that these auxin-related genes could enhance pepper’s to promote plant cell division and differentiation. Furthermore, previous studies revealed that *IAA* might interact with different ARFs, resulting in transcriptional inhibition or activation (Su et al., 2014). These results show that the callus induction process is based on the synergistic effect of phytohormones.

### Analysis of starch and sucrose metabolism in callus induction

Sucrose is the main carbohydrate supplement in the culture media. It plays an important role as an energy source and osmosis in the process of organogenesis (Verma and Dougall, 1977; Cui et al., 2019). Moreover, the correlation between starch metabolism and shoot regeneration has been reported in rice (Lee and Huang, 2013; Lee and Huang, 2014) and sorghum (Zhou et al., 2021). Previous studies indicated that SWEET13 and SWEET14 proteins were linked to sugar transport and involved in gibberellin-mediated physiological processes at the seedling stage of *Arabidopsis* (Kanno et al., 2016). In the present study, *SWEET14* was identified as a hub gene in the network. It may be related to the sucrose metabolic pathway. Furthermore, the majority of genes regulated by starch and sucrose were up-regulated in HNUCB81 and HNUCB226 compared with HNUCC22 and HNUCC16. The results revealed that *C. baccatum* expression was significantly higher than *C. chinense* during S2 and S3. The findings suggest that these genes may regulate the metabolism of starch and sucrose and play a key role in the process of regeneration.

### Roles of *WOX* gene in the regeneration of pepper

*WOX* family genes are important in a variety of processes, ranging from callus formation to organ development (Kim et al., 2018; Wang C. et al., 2021; Willoughby and Nimchuk, 2021). It was reported that *WOX2* and *WOX8*/9 were involved in differentiation and somatic embryogenesis (Palovaara et al., 2010; Zhao et al., 2022). Based on the activity of *WIND1*, *WOX13* was rapidly induced, which is a key regulator of callus formation (Ikeuchi et al., 2021). In addition, previous studies found that *WOX7* inhibited lateral root initiation and primordium growth (Kong et al., 2016). In contrast, we have shown rapid increases in *WOX7* expression in *C. baccatum* lines between S2 and S3 but it is down-regulated in *C. chinense* lines. Interestingly, *WOX7* was a significant expression within the WGCNA module, consistent with RT-qPCR. It was also found that *WOX* genes were up-regulated in the high-regeneration lines of maize (Zhang et al., 2019). These results demonstrated that *WOX* could reflect a positive function in regeneration.

### Hub genes involved in regeneration

Several hub genes have been identified *via* network analysis that may be key regulating factors in regeneration. Previous studies have indicated that some WRKY genes induce callus of papaya and *Panax ginseng* (Jamaluddin et al., 2017; Yang et al., 2020). Moreover, WRKY genes may upregulate callus formation in bread wheat (Chu et al., 2017). In our study, WRKY TFs were up-regulated between HNUCB226_S3 *vs* HNUCB226_S2, but there was no significant expression in *C. chinense*. These results revealed that WRKY TFs play an important role in the process of regeneration.

Furthermore, several callus-related genes, including *GRF, GDSL, TSJT1*, and *CCCH*, have been identified as being associated with dedifferentiation (Zhang et al., 2022). Our results indicated that these hub genes were highly expressed in HNUCB81 and HNUCB226, but without fluctuation in *C. chinense*. In addition, it is reported that the ABC transport family mediates the transport of auxin in roots to promote basipetal transport (Ko et al., 2014). Interestingly, we identified a hub gene for *ABCB7* in the lightcyan module, suggesting its essential role in initial callus induction.

## Conclusion

A comprehensive understanding of the genes associated with pepper callus development will help to understand the process of pepper regeneration. In the present study, we successfully established an efficient *in vitro* regeneration system in two cultivars to monitor callus induction in differential genotypes. In addition, we performed histology and transcriptome analysis of the pepper regeneration and demonstrated a high correlation between somatic callus induction and plant hormone pathway and starch and sucrose metabolism pathways. This study provides novel information for the induction of regeneration and will have great potential for transgenic plants or molecular-assisted breeding of pepper.

## Materials and methods

### Plant materials and construction of regeneration

We kept *Capsicum baccatum* (HNUCB81 and HNUCB226) and *Capsicum chinense* (HNUCC22 and HNUCC16) on bud induction media (B1-B12), which was supplemented with 4.44 - 22.2µM (6-BA) and 0.57 - 2.85µM IAA (**Supplementary Table S1**). We also kept on the plant elongation media (E1-E12), which was supplemented with 13.32 – 22.2µM (6-BA) + 2.85-5.71µM IAA + 0 – 11.55µM GA_3_ (**Supplementary Table S2**), rooting media (R1-R6) supplemented with 0.57 - 2.85µM IAA or 0.54 - 2.69µM NAA mg · L^−1^ (**Supplementary Table S3**). MS media including 6 g/L agar and 30g/L sucrose (Hopebio, Qingdao) and phytohormone (Sigma, St. Louis, MO, USA). Each treatment contained at least 30 explants derived from petiole of cotyledons of sterile seedling aged 15-20 days (0.5-1cm in length). All media were adjusted to pH 5.8 and then autoclaved at 116°C for 30 minutes. The explants were inoculated on B media for 15-20 d to get the plantlets, and after elongation incubated in E media to proliferate buds, and was transplanted to R media to induct roots. All of the explants were cultured in MS media at 24 ± 2°C with 16 h light/d for differentiation at Hainan University. All experiments were repeated three times. The measurement of data was calculated with the equation: percentage of callus induction: Callus induction (%) = No. of Callus/No. of explants ×100%; Plantlets induction (%) = No. of plantlets/No. of Callus ×100%; Rooting induction (%) = No. of rooting plants/No. of plantlets ×100%. Statistical analysis was done using IBM SPSS (v26). The significance of the difference between the mean values was tested using Duncan’s multiple range test (P ≤ 0.05). The results are indicated as mean ± standard error of three duplications.

### Histological analysis

Based on the regeneration results, we collected early differentiated callus supplemented with 13.32µM 6-BA+ 2.85µM IAA at three different stages, including S1 (1-3d), S2 (4-6d), and S3 (7-9d). The callus tissues were collected in a 50% FAA solution. After fixation, the slices were dehydrated successively with xylene and ethanol. After that, the slices were dyed in Safranin O-Fast Green staining solution. Moreover, slices were placed in clean xylene and stuck tissue slices with neutral balm (Servicebio, Wuhan). Finally, the slices were observed and photographed under the microscope (Nikon Eclipse E100 and Nikon DS-U3). Triplicate biological replicates for each sample.

### Sequencing and mapping analysis

We selected explants with normal development (No contamination and browning) for mixed sampling at three early development stages, including S1 (1-3d), S2 (4-6d), and S3 (7-9d). Total RNA was isolated with Trizol Reagents (Thermo Fisher Scientific, Shanghai, China). Thirty-six non-directional libraries were produced using the NEBNext^®^ Ultra™ RNA Library Prep Kit for Illumina^®^ (NEB, United States) and were sequenced on the Illumina Novaseq platform. Clean reads were obtained by filtering low-quality reads and aligned to the *C. chinense* reference genome (https://www.ncbi.nlm.nih.gov/genome/?term=Capsicum+chinense) using HISAT2 (v.2.0.5) (Kim et al., 2015).

### Differential gene expression statistics

The fragments per kilo-base of exon per million fragments mapped (FPKM) was to estimate the transcript expression level of all samples, which was calculated by StringTie (v1.3.3b) (Pertea et al., 2016). Significantly differential expression genes (DEGs) were identified by DESeq2 R package (v1.20.0) (Love et al., 2014) with a threshold included |log2 (Foldchange)| > 1 and *p*-adjust < 0.05. The Venn diagram of the DEGs was created using Jvenn (Bardou et al., 2014), and the heat map was plotted using the R package of pheatmap.

### Annotation and functional classification

Differential expression genes enrichment analysis of Gene Ontology (GO) was applied by GOseq (v1.22) software (Young et al., 2010). The setting parameter is that the *p-* adjust < 0.05. Various metabolic pathways were identified based on the Kyoto Encyclopedia of Genes and Genomes (KEGG) database. The enrichment analysis of DEGs was calculated by KOBAS 2.0 software (Mao et al., 2005). *p-*adjust < 0.05 was distributed to be significantly enriched in KEGG.

### WGCNA gene coexpression network

WGCNA is applied to analyze gene expression patterns in different samples to obtain gene modules with similar expression patterns (Langfelder and Horvath, 2008). In this WGCNA network, the soft power was selected at 10 (*R* ^2 =^ 0.8), mergeCutHeight was set as > 0.75, and minModuleSize was set as > 30. WGCNA network was constructed by WGCNA in the R package and the genes from each module were visualized using Cytoscape (v3.8.0).

### Quantitative real-time PCR validation

Total RNA was subjected to reverse transcription using Hiscript III RT SuperMix for qPCR (Vazyme Biotech, China), and qPCR was analyzed using SYBR qPCR Master Mix (Vazyme Biotech, China). The amplification program was based on the standard protocol of the Applied Biosystems QuantStudio 1 Real-Time PCR Instrument (Thermo Fisher Scientific, USA), as follows 95°C for 10 min, 40 cycles of 95°C for 15s, and 60°C for 10s, and thermal denaturation step is then performed to generate a melting curve to verify the amplification specificity. The primers were designed by primer 5 (v5.0) that were listed in **Supplementary Table S9**.

## Data availability statement

The original contributions presented in the study are publicly available. This data can be found here: NCBI, PRJNA790105.

## Author contributions

For research articles: Conceptualization, ZW, HS; methodology and validation, HS; investigation, YZ, CH, MA, YH, DL, LL, CL, FH, SC, and GZ; writing—original draft preparation, HS; supervision, ZW. All authors have read and agreed to the published version of the manuscript.

## Funding

This work was supported by a grant from the Project of the Administrative Bureau of Sanya Yazhou Bay Science and Technology City (HNF202210), the Major Science and Technology Plan of Hainan Province (ZDKJ2021010) and the National Key Research and Development Program of China (2018YFD1000800).

## Acknowledgments

We thank Dr. Muhammad Ali Mumtaz and Dr. Sunjeet Kumar from School of Horticulture for their suggestions on the manuscript.

## Conflict of interest

The authors declare that the research was conducted in the absence of any commercial or financial relationships that could be construed as a potential conflict of interest.

## Publisher’s note

All claims expressed in this article are solely those of the authors and do not necessarily represent those of their affiliated organizations, or those of the publisher, the editors and the reviewers. Any product that may be evaluated in this article, or claim that may be made by its manufacturer, is not guaranteed or endorsed by the publisher.
