# Supplementary material for: Establishment of in vitro regeneration system and molecular analysis of early development of somatic callus in Capsicum chinense and Capsicum baccatum
Source: Front Plant Sci. 2022 Nov 17;13:1025497. doi: 10.3389/fpls.2022.1025497 (PMC9714296; doi:10.3389/fpls.2022.1025497)
Supplement: Supplementary file 10 [file DataSheet_1.docx]

***Supplementary Material***

**Supplementary Figure S1.** Results of gene quantitative analysis. (A) Correlation coefficient analysis of each sample. (B) Principal component analysis (PCA) of each sample.

**Supplementary Figure S2.** Number of DEGs. (A) Different genes in different samples. (B) Venn diagram of the DEGs for each genotype.

**Supplementary Figure S3.** Comparison of Gene Ontology (GO) classification of DEGs at the three modules. (A) lightcyan module; (B) lightyellow module; (C) midnightblue module.
